# Recent advances in understanding immune phenotypes of thyroid carcinomas: prognostication and emerging therapies

**DOI:** 10.12688/f1000research.16677.1

**Published:** 2019-02-28

**Authors:** Federica Liotti, Nella Prevete, Giancarlo Vecchio, Rosa Marina Melillo

**Affiliations:** 1 Dipartimento di Medicina Molecolare e Biotecnologie Mediche, Università di Napoli Federico II, Naples, Italy, Italy; 2 Dipartimento di Scienze Mediche Traslazionali, Università ¨Federico II¨ di Napoli, Naples, Italy, Italy; 3 Istituto di Endocrinologia e Oncologia Sperimentale “G. Salvatore”, CNR, Naples, Italy, Italy; 4 Istituto Superiore di Oncologia, Naples, Italy; 5 Istituto Superiore di Oncologia, Genoa, Italy

**Keywords:** papillary thyroid carcinomas, Immunotherapy, Immune Checkpoint

## Abstract

Tumors modulate the host immune cells within their microenvironment to avoid recognition and elimination by our immune system, a phenotype called cancer immune escape. Different mechanisms responsible for cancer immune escape that result either in decreased tumor immunogenicity or in increased tumor immunosuppressive activity have been identified. Recently, various immunotherapeutic approaches have been developed with the aim to revert tumor immune escape. The aims of this review are to explore the immunological aspects of thyroid cancer and to assess whether these features can be exploited in the prognosis and treatment of advanced forms of this disease. Therefore, we will describe the immune landscape and phenotypes of thyroid cancer, summarize studies investigating the expression of immunomodulatory molecules, and finally describe the preclinical and clinical trials investigating the utility of immunotherapies in the management of thyroid cancer.

The aim of this review is to explore the immunological aspects of thyroid cancer and to assess whether these features can be exploited in the prognosis and treatment of advanced forms of this disease. Therefore, we will describe the immune-landscape and phenotypes of thyroid cancer, we will summarize studies investigating the expression of immunomodulatory molecules, and we will finally describe the preclinical and clinical trials investigating the utility of immunotherapies in the management of thyroid cancer.

## Immune escape: a new hallmark of cancer

In 2011, Hanahan and Weinberg published a seminal review on the “Hallmarks of cancer” summarizing the features that a normal cell must acquire in order to become cancerous ^1, 2^. The classic traits include intrinsic features of the cancer cell, such as cell autonomous growth and resistance to apoptosis, as well as characteristics linked to the tumor microenvironment (TME), such as invasive ability and angiogenic potential. Moreover, other features, including the ability of neoplastic cells to evade the anti-cancer immune response, have been identified ^1– 3^.

During tumor evolution, the escape of cancer cells from immune surveillance has been proposed to occur in three phases: elimination, equilibrium, and escape ^4^.

- In the elimination phase, newly transformed cells can be recognized and eliminated by an immune response, evoked by natural killer (NK) and other immune cells.
- In the equilibrium phase, the selective pressure exerted by the immune system leads to the elimination of the most immunogenic cancer cell clones, allowing less immunogenic cells to survive.
- In the escape phase, cancer cell clones emerge that are resistant to immune attack, thus allowing tumor progression and metastasis.

Thus, it has become clear that the immune system plays a central role in tumor biology and that, despite the presence of an anti-cancer immune response, tumors often activate various mechanisms in order to escape from immune-mediated elimination.

## How tumors can avoid immune recognition/elimination

Cancer cells can be recognized and eliminated by our immune system. Innate and adaptive immune effector mechanisms contribute to tumor recognition and control. First, tumor cells are recognized by NK cells which participate in the destruction of transformed cells. Tumor cell fragments are then taken up and processed by macrophages and dendritic cells, which in turn secrete inflammatory cytokines and present tumor antigens to T cells. Activation of T and B cells promotes the expansion of tumor-specific T cells and antibodies ^5^.

Tumors are able to modulate the host immune cells within the TME to escape their surveillance. This can occur by recruiting immunosuppressive cells, by decreasing tumor immunogenicity, or by exploiting immunosuppressive mechanisms ^6^.

Many tumors display immune infiltrating cells, similar in phenotypes to those recruited during the resolution phase of wound healing ^7^ wherein the tissue microenvironment is enriched of leukocytes with immunosuppressive properties. This immunosuppressive context causes a block of cytotoxic T lymphocyte- or NK-mediated killing of transformed cells ^8^. Furthermore, myeloid-derived suppressor cells (MDSCs) and tumor-associated macrophages (TAMs) polarized toward an M2 phenotype are present and can contribute to cancer escape from immune killing. In addition to the above-mentioned cells, often regulatory T cells (Tregs) are enriched within primary and metastatic tumors. Tregs are CD4 ^+^/CD25 ^+^ T lymphocytes associated with poor prognosis for cancer ^9^. Tregs specifically exert their immunosuppressive functions via the production of soluble factors - that is, interleukin-10 (IL-10), transforming growth factor beta (TGFβ), and IL-35 - or via cell contact inhibiting the activation of the T-cell receptor (TCR) ^10^. Consistently, targeting of Tregs improves tumor immunity, providing a clinical benefit in many cancer types ^11^.

In order to escape immune-mediated elimination by tumor-specific T cells, cancer cells may decrease their antigenicity by downregulating the expression of tumor antigens ^12^ or may impair their capacity to present antigens by losing major histocompatibility complex (MHC) I expression. Alternatively, tumors that retain sufficient antigenicity for immune recognition can escape elimination by upregulating both soluble or membrane-associated immune-inhibitory molecules ^13^.

Among the most important inhibitory molecules, crucial for the physiologic maintenance of self-tolerance, are the co-inhibitory receptors cytotoxic T-lymphocyte antigen 4 (CTLA-4), programmed cell death-1 (PD-1), and programmed cell death-ligand 1 (PD-L1) and 2 (PD-L2), also defined as immune checkpoints. Cancer cells exploit immune checkpoints to inhibit the anti-tumor immune response.

Not only membrane receptors are responsible for immunoresistance of tumors. In some cases, soluble factors also may shape the TME contributing to the anti-cancer immune response suppression. The production of metabolic enzymes such as arginase (ARG) and indoleamine 2,3-dioxygenase (IDO) or of immunosuppressive cytokines (for example, TGFβ and IL-10) can determine an inhibitory context for anti-tumor T cells ^14– 16^.

## Immunotherapy in cancer: the most promising approaches

Based upon the above-mentioned notions, the use of the immune system as a tool to destroy cancer cells appears to be a very promising approach. Various “immunotherapeutic” strategies can be applied to reach this goal. One possible approach is to revert tumor-associated immune suppression, thus favoring the reactivation of the cytotoxic anti-cancer immune responses. On the other hand, increasing tumor immunogenicity by stimulating the expression of MHC molecules or by promoting the expression of tumor-associated antigens might be another strategy to elicit an efficient anti-cancer immunity. Finally, vaccination with tumor antigens or adoptive transfer of activated immune cells might be used as a strategy to boost anti-cancer immunity ^17^.

Among the different immunotherapeutic approaches to cancer treatment, the most promising are summarized here:

- Immune checkpoint inhibitors (ICIs). This approach is based upon the use of neutralizing monoclonal antibodies directed to immune checkpoints. These treatment options are emerging as promising treatments for various types of cancers as they are able to block the co-inhibitory signals, thus re-activating a prolonged anti-tumor response. The US Food and Drug Administration (FDA) has recently approved several ICIs: the anti-CTLA-4 ipilimumab for the treatment of melanoma; the PD-1 blocking antibodies pembrolizumab and nivolumab for unresectable or metastatic melanoma, non-small cell lung cancer (NSCLC), head-and-neck squamous cell carcinoma, Hodgkin’s lymphoma, and for bladder and renal cell carcinoma; the anti-PD-L1 atezolizumab has been approved for metastatic bladder cancer and NSCLC. However, additional clinical indications for ICIs are under consideration in many clinical trials ^18, 19^.
- Personalized vaccines. So far, many vaccination approaches, including immune cell– or dendritic cell–based, tumor cell–based, oncolytic viruses, nanocarrier-delivered, and DNA-based vaccines, have been developed ^20, 21^. These treatments can be administered to patients to induce host immunity against specific tumor antigens. The use of a vaccine has the aim of encouraging the body to develop an immune response targeting tumor-specific antigens. Personalized vaccines are developed starting from patient biopsies sequenced through next-generation sequencing. Then cancer and normal genomes are compared, and the most immunogenic epitopes are identified by using bioinformatics tools. Finally, the vaccine is developed as a poly-protein or a poly-mRNA and administered to the patient. Both preclinical and clinical studies provided solid evidence that personalized cancer vaccines could exert powerful neoantigen-specific T-cell responses against different cancer types ^22^.
- Chimeric antigen receptor T (CAR T)-cell therapy. CAR T-cell therapy has been developed to target tumor-specific antigens using an engineered CAR containing the antigen-specific binding domain of an antibody fused to the TCR and CD28 intracellular portions retaining the signaling domains. The great advantage of this approach is that the cytotoxic function of CAR T cells does not depend on the MHC presentation of tumor antigens and does not need co-stimulation. CAR T approaches showed remarkable success in acute B-cell leukemia with up to 90% of complete remission. CAR T-cell therapy has been approved by the FDA and by the European drug agency (European Medicines Agency) for different types of lymphoma and leukemia. A broad spectrum of CAR T-cell therapies are now being tested in clinical trials for solid tumors ^23, 24^.

## Thyroid cancer: a candidate for immunotherapy?

Papillary thyroid carcinoma (PTC) is the predominant histologic type of thyroid cancers (TCs), representing around 90% of all cases. It is usually well treatable with an optimum 10-year survival rate (>90%) ^25^. Surgery is the definitive treatment option for most PTCs, followed eventually by radioiodine (RAI) therapy in the high-risk group. However, some PTCs (5–10%) experience recurrence and distant metastasis and fail to respond to RAI therapy. These tumors, together with poorly differentiated TCs (PDTCs) and anaplastic thyroid carcinomas (ATCs), are considered “advanced thyroid cancers”. These carcinomas represent a therapeutic challenge because they are more difficult to cure with current therapies.

Multiple kinase inhibitors (MKIs) targeting receptor tyrosine kinase (RTK) or the mitogen-activated protein kinase (MAPK) signaling pathways are used to treat RAI-refractory TCs. These treatments exert cytostatic effects on TC cells but can also achieve (RAI) re-sensitization ^26^. However, the development of MKI tumor resistance mechanisms has limited their clinical benefits ^27^.

The aims of this review are to explore the immunological aspects of TCs and to assess whether this knowledge can aid in the prognosis and treatment of advanced TCs. Therefore, we will first describe the immune landscape and phenotypes of TCs, summarize studies investigating immune checkpoint expression in TCs, and finally describe the preclinical and clinical trials investigating the utility of immunotherapies in the management of TCs.

### Immune landscape of thyroid carcinomas

The evaluation of the immunological features of TCs is crucial to understand the specific patterns of immune cells infiltrating TCs, their phenotypes, and their functional significance in the context of thyroid neoplastic lesions. The analysis of transcriptomic and genomic data already available in The Cancer Genome Atlas, by applying and integrating major immunogenomics methods, was useful to characterize the immune components of TME and to classify tumors into six types based upon their “immunoscore”. Via evaluation of the immunoscore, PTCs have been classified as “inflammatory” tumors ^28^. PTCs are tumors with low mutational burden due to low neoantigen expression, which is suggestive of poor immunogenicity. However, they display an important immune infiltrate that can account for the “inflammatory” immunoscore. Whether PTC-associated inflammation is due to some intrinsic features of the thyroid gland, including the presence and abundance of tissue-specific antigens, or to the frequent disruption of immunological tolerance and the consequent propensity for autoimmunity is still a matter of debate. Whatever the case, the presence of autoimmunity or of chronic lymphocytic thyroiditis has been correlated with a favorable prognosis both in TC and in other cancer types ^29, 30^. On the contrary, immunosuppressive cell populations have also been described in PTCs and their density often correlated with a poor prognosis.

Several immune cells infiltrating TC have been described as being potentially involved in anti-tumor immune response:

- Tregs exert a pro-tumorigenic function in PTCs by expressing immune checkpoints. They are enriched in localized and advanced TCs, lymph nodes, and metastasis. Thus, it is clear that their presence correlates with tumor aggressiveness ^31, 32^.
- Patients with advanced forms of TC display a low number of peripheral blood NK cells in comparison with those of control patients. In a murine model of TC, the introduction of the NK activating cytokine IL-12 restored the anti-tumor immune response ^33, 34^.
- In PTCs and PDTCs, a great density of M2 TAM with a tumor-promoting and immuno-suppressive phenotype has been observed. Their number correlates with a dismal prognosis and a decreased survival ^35– 37^.
- Dendritic cells with an immature phenotype are present in PTCs. This phenotype is associated with a reduced efficiency to present antigens and to sustain an efficient immune response to cancer cells ^38^.
- Mast cells are enriched in PTCs compared with normal thyroid tissues. Their functional role in sustaining TC proliferation, invasion, stemness, and aggressiveness through the production of inflammatory mediators (that is, CXCL1, CXCL8/IL-8, and CXCL10) has been largely described ^39– 41^.

Immunoscores have also been correlated with specific genetic lesions and with the differentiation score in TC. For instance, Na and Choi found that enrichment scores for dendritic cells, macrophages, and mast cells in PTCs correlated with low thyroid differentiation score or with BRAF ^V600E^ mutation ^42^. The same study demonstrated that the expression levels of CTLA-4 and PD-L1 were higher in BRAF ^V600E^-positive and in de-differentiated TCs ^42^. These results are consistent with previous immunohistochemical (IHC) results showing that the BRAF ^V600E^ status was closely associated with Tregs and immunosuppressive macrophage components as well as with immunosuppressive markers, including PD-L1 ^43^.

Higher levels of PD-L1 expression were also found in PTCs to correlate with TAM and CD8 ^+^, CD4 ^+^, and Treg lymphocytic infiltrate ^44, 45^. Furthermore, in PDTCs, the expression of PD-L1 was significantly associated with increased tumor size and multifocality. In metastatic forms of PTCs, PD-1 ^+^ T-lymphocytes were found in lymph nodes, showing a significant association with cancer lymph-nodal invasion and recurrent disease ^46^.

The immuno-suppressive context observed in TCs is sustained also by IDO production by tumor cells as demonstrated in several reports. In particular, IDO expression in TCs was associated with an increased Treg infiltrate and with more aggressive clinic-pathologic features, such as extra-thyroidal extension or multifocality ^47, 48^.

### Immunotherapy for advanced thyroid carcinomas

Several reports point to a promising role of immunotherapy in the treatment of advanced forms of TCs. Here, we will briefly discuss the utility of CAR T and vaccines and we will focus on clinical trials evaluating ICIs alone or in combination with other pharmacologic approaches in the treatment of advanced TCs.

***Chimeric antigen receptor T***. Min *et al*. evaluated the potential benefit of a CAR T approach targeting intercellular adhesion molecule 1 (ICAM-1) expressed on the surface of primary and metastatic PTCs and ATCs ^49^. The authors demonstrated that CAR T therapy represents a therapeutic strategy providing a significant survival benefit in animal models. This report demonstrated for the first time the utility of a CAR T-cell therapy for advanced TCs in a preclinical setting, opening the possibility to develop other CAR T approaches targeting membrane antigens highly expressed by TCs, such as human epidermal growth factor receptor 2 (HER2) ^49^.

***Vaccines***. The identification of neoantigens or tumor-specific antigens is crucial for the development of vaccines using dendritic cells or cancer-specific T cells. The potential use of thyroglobulin (Tg) as TC-specific antigen is under investigation in clinical trial NCT02390739. In this protocol, patient-derived T cells from differentiated TCs are transduced with a TCR recognizing Tg-derived epitopes and administered, together with recombinant human IL-2, to favor T-cell proliferation ^50, 51^. Other neoantigens are under study as targets of cancer vaccines for solid tumors, including those derived from advanced TCs. One of these trials is NCT02239861, investigating cytotoxic T cells targeting several tumor antigens (NY-ESO-1, MAGEA4, PRAME, survivin, and SSX) ^52^.

***Immune checkpoint inhibitors.*** In keeping with the increased expression of immune checkpoints in advanced TCs compared with differentiated forms and with their correlation with a negative prognosis, few case reports suggest the potential benefits of ICIs in patients with TC. In particular, it has been demonstrated that pembrolizumab (anti-PD-1) alone can be used as a neoadjuvant approach enabling the complete surgical resection of a BRAF-mutated ATC. Furthermore, in a subset of patients with ATC, pembrolizumab was shown to be an effective salvage therapy added to kinase inhibitors ^53, 54^. Several clinical trials, summarized in Table 1, are now investigating the utility of ICIs in monotherapy or combination therapy for advanced TCs.

**Table 1.** Ongoing clinical trials testing immune checkpoint inhibitors in thyroid cancer.

Trial number (ClinicalTrials.gov Identifier)	Drug(s)	Brief description	Completion date	Phase	Number of participants	Type
NCT02054806	Pembrolizumab	Assessing the efficacy and safety of pembrolizumab in participants with incurable advanced solid tumors that have not responded to current therapy	8/13/2019	Ib	477	Interventional, single-group assignment
NCT02628067	Pembrolizumab	Assessing the efficacy of pembrolizumab in participants with multiple types of advanced (unresectable and/or metastatic) solid tumors that have progressed on standard-of-care therapy	8/28/2023	II	1350	Interventional, single-group assignment
NCT03246958 II	Nivolumab + ipilimumab	Studying nivolumab in combination with ipilimumab as a possible treatment for thyroid cancer	3/31/2025	II	54	Interventional, parallel assignment
NCT02501096	Levantinib + pembrolizumab	Assessing the maximum tolerated dose for levantinib in combination with pembrolizumab in phase Ib. Evaluating the safety and efficacy of this combination in phase II for several solid tumors	2/29/2020	Ib/II	329	Interventional, single-group assignment
NCT02973997	Levantinib + pembrolizumab	Studying the efficacy of pembrolizumab + lenvatinib in patients with differentiated thyroid cancer that has spread to other places in the body or has come back and cannot be removed by surgery	9/30/2022	II	60	Interventional, single-group assignment
NCT03181100	Atezolizumab + targeted therapy or taxanes	Assessing the effects and safety of targeted therapy + atezolizumab or taxanes + atezolizumab in patients with anaplastic thyroid carcinoma and poorly differentiated thyroid carcinoma	7/2/2023	II	50	Interventional, non-randomized
NCT01988896	Atezolizumab + cobimetinib	Testing the effects of combining atezolizumab + cobimetinib in locally advanced or metastatic solid tumors	4/19/2019	Ib	153	Interventional, non-randomized

Clinical trial NCT02054806 is a non-randomized trial testing pembrolizumab (anti-PD-1) in 20 different types of solid tumors, including 22 patients with papillary subtypes of advanced TCs. To date, it has been reported that 2 out of 22 patients had a partial response while 12 out of 22 patients displayed stable disease; 18 out of 22 patients developed treatment-related side effects, although none of them discontinued the treatment or died for adverse effects ^55^. Thus, pembrolizumab showed potential anti-tumor effects in advanced PTCs. However, the results of this protocol have to be further investigated in phase 2 of the trial (NCT02628067) ^56^.

In addition, the possibility of combining two ICIs - nivolumab (anti-PD-1) plus ipilimumab (anti-CTLA-4) - is under investigation in an ongoing trial (NCT03246958 II) in metastatic RAI-resistant TCs.

Interestingly, the possibility of combining ICIs with currently available drugs for advanced TCs has attracted attention. In particular, it has been noted that kinase inhibitors have the ability to induce immune modulation; thus, several clinical trials are investigating the possibility of combining ICIs and kinase inhibitors to achieve a clinical response in advanced TCs.

NCT02501096 is a phase IB/II trial with the aim of defining the maximum tolerated dose for lenvatinib - a potent inhibitor of vascular endothelial growth factor (VEGF) receptors, fibroblast growth factor receptors, platelet-derived growth factor receptor alpha, c-KIT, and rearranged during transfection (RET) - in combination with pembrolizumab in patients with several types of solid tumors, including advanced TCs ^57^. A trial started in April 2017 (NCT02973997) is investigating pembrolizumab plus lenvatinib in patients with locally recurrent and unresectable or distant metastatic TCs (or both) to evaluate the safety and efficacy of this combination ^58^. An ongoing trial (NCT03181100) has the primary objective of determining the effects of several targeted therapies plus atezolizumab on overall survival (OS) of patients with ATC; its secondary objectives are to evaluate the safety and efficacy of targeted therapy plus atezolizumab in ATCs and PDTCs, to determine the OS in patients with PDTCs treated with targeted therapy plus atezolizumab, and to determine the OS of patients with ATC and PDTC treated with taxanes plus atezolizumab ^59^. A similar protocol (NCT01988896) will test the effect of the combination of atezolizumab (anti-PD-L1) plus cobimetinib (MAPK inhibitor) in advanced or metastatic solid tumors, including TCs ^60^.

## Conclusions

Advanced TCs have limited treatment options ^61^. In fact, once they acquire RAI resistance, TCs are generally treated by cytotoxic drugs with high toxicity or by targeted therapies with development of resistance.

The promising results of ICIs have accelerated and improved research assessing the efficacy of these drugs in many cancer types, including advanced TCs. Thyroid neoplasias are increasingly being addressed by using ICIs, and several ongoing clinical trials are being conducted on patients with TCs. We believe that strategies targeting immunity, possibly in combination with other therapies, are likely to achieve durable responses in these neoplasias.

## Abbreviations

ATC, anaplastic thyroid carcinoma; CAR T, chimeric antigen receptor T; CTLA-4, cytotoxic T-lymphocyte antigen 4; FDA, US Food and Drug Administration; ICI, immune checkpoint inhibitor; IDO, indoleamine 2,3-dioxygenase; IL, interleukin; MAPK, mitogen-activated protein kinase; MHC, major histocompatibility complex; MKI, multiple kinase inhibitor; NK, natural killer; NSCLC, non-small cell lung carcinoma; OS, overall survival; PD-1, programmed cell death-1; PD-L1, programmed cell death-ligand 1; PDTC, poorly differentiated thyroid carcinoma; PTC, papillary thyroid carcinoma; RAI, radioiodine; TAM, tumor-associated macrophage; TC, thyroid cancer; TCR, T-cell receptor; Tg, thyroglobulin; TGFβ, transforming growth factor beta; TME, tumor microenvironment; Treg, regulatory T cell
